# p32 is a negative regulator of p53 tetramerization and transactivation

**DOI:** 10.1002/1878-0261.12543

**Published:** 2019-07-30

**Authors:** Nikhil Baban Ghate, Jinman Kim, Yonghwan Shin, Alan Situ, Tobias S. Ulmer, Woojin An

**Affiliations:** ^1^ Department of Biochemistry and Molecular Medicine, Norris Comprehensive Cancer Center University of Southern California Los Angeles CA USA; ^2^ Department of Biochemistry and Molecular Medicine, Zilkha Neurogenetic Institute University of Southern California Los Angeles CA USA

**Keywords:** nuclear export signal, p32, p53, tetramerization, transcription

## Abstract

p53 is a sequence‐specific transcription factor, and proper regulation of p53 transcriptional activity is critical for orchestrating different tumor‐suppressive mechanisms. p32 is a multifunctional protein which interacts with a large number of viral proteins and transcription factors. Here, we investigate the effect of p32 on p53 transactivation and identify a novel mechanism by which p32 alters the functional characteristics of p53. Specifically, p32 attenuates p53‐dependent transcription through impairment of p53 binding to its response elements on target genes. Upon p32 expression, p53 levels bound at target genes are decreased, and p53 target genes are inactivated, strongly indicating that p32 restricts p53 occupancy and function at target genes. The primary mechanism contributing to the observed action of p32 is the ability of p32 to interact with the p53 tetramerization domain and to block p53 tetramerization, which in turn enhances nuclear export and degradation of p53, leading to defective p53 transactivation. Collectively, these data establish p32 as a negative regulator of p53 function and suggest the therapeutic potential of targeting p32 for cancer treatment.

**Abbreviations**
Acf1: ATP‐utilizing chromatin assembly factor 1
GST: glutathione *S*‐transferase
HA: hemagglutinin
NES: nuclear export signal
p53RE: p53 response element
qPCR: quantitative PCR
RT‐qPCR: real‐time quantitative PCR
TFIIB: transcription factor IIB

## 1 Introduction

p32, also known as gC1qR/C1QBP/HABP1, was first identified as a factor that is associated with splicing factors and is required for 5′ splice site cleavage and lariat formation during pre‐mRNA splicing in HeLa cells (Krainer *et al.*, 1990). Initial analysis detected p32 in the mitochondrial matrix, but p32 was also reported to be present in the nucleus in later studies, suggesting that p32 can shuttle between the mitochondria and the nucleus depending on its function (Matthews and Russell, 1998). p32 exists in an equilibrium between monomers and trimers which retain specific binding affinities to other proteins (Jha *et al.*, 2002; Jiang *et al.*, 1999). The crystal structure of p32 was solved, revealing that p32 can form a doughnut‐shaped trimer with the negatively charged residues asymmetrically distributed on one face and lining the channel of the complex (Jiang *et al.*, 1999). p32 was shown to have the ability to interact with several viral proteins including core protein V of adenovirus, ORF P of herpes simplex virus, EBNA‐1 of Epstein–Barr virus, and HIV‐1 Tat and Rev (Bruni and Roizman, 1996; Desai *et al.*, 1991; Fridell *et al.*, 1995; Luo *et al.*, 1994; Matthews and Russell, 1998; Tange *et al.*, 1996; Wang *et al.*, 1997; Yu *et al.*, 1995; Yu *et al.*, 1995). These results suggest its possible importance in the regulation of replication and generation of viral particles. p32 was also reported to bind the C‐terminal region of the general transcription factor IIB (TFIIB), which led to the proposal that p32 may be directly involved in transcription by acting as a bridging factor between TFIIB and transcriptional activators (Yu *et al.*, 1995; Yu *et al.*, 1995). Adding to the list of diverse binding partners, the interaction of p32 with lamin B receptor, high molecular weight kininogen and factor XII, plasma complement component C1q, vitronectin, and hyaluronic acid was also reported (Deb and Datta, 1996; Ghebrehiwet *et al.*, 1994; Herwald *et al.*, 1996; Lim *et al.*, 1996; Simos and Georgatos, 1994). From a functional point of view, p32 is implicated in maintaining mitochondrial oxidative phosphorylation (Muta *et al.*, 1997) as well as mediating ARF‐induced apoptosis (Itahana and Zhang, 2008). A strong support for an oncogenic function of p32 is also provided by studies demonstrating that cancer cells generally express higher levels of p32 compared to their normal counterparts (Chen *et al.*, 2009; Fogal *et al.*, 2008; Rubinstein *et al.*, 2004).

The tumor suppressor p53 plays an important role in the cellular response to various stresses such as DNA damage, hypoxia, and aberrant oncogene signals. Although some p53 effects may involve nontranscriptional mechanisms, many roles played by p53 are mediated through its function as a transcription factor that induces specific gene transcription programs (Jiang *et al.*, 2010; Murray‐Zmijewski *et al.*, 2008; Speidel *et al.*, 2006). p53 can be divided into four major domains depending on their structure and function: the N‐terminal transactivation domain (1–80 residues), the central DNA‐binding domain (100–300 residues), the tetramerization domain (323–355 residues), and the C‐terminal regulatory domain (364–393 residues) (Joerger and Fersht, 2007; Wang *et al.*, 1994). In unstressed cells, p53 exists as a monomer that is constitutively ubiquitinated and rapidly degraded via ubiquitin proteasome pathways in the cytoplasm (Brooks and Gu, 2006). Upon DNA damage, p53 is stabilized and activated to form a tetrameric complex in which the nuclear export signal (NES) is blocked, ensuring that the active tetramer is retained in the nucleus where, with the help of C‐terminal regulatory domain, it acts on target genes (Prives and Hall, 1999; Sullivan *et al.*, 2018). Thus, the tetramerization is one of the primary mechanisms responsible for p53 stabilization and its transactive function in response to DNA damage. Cancer cells developed several strategies to escape from p53‐mediated stress response, one of which is the inhibition of tetramer formation. In this regard, several mutations in the tetramerization domain of p53 have been reported to compromise p53 tetramer formation and thereby inhibit DNA binding and transactivation activity (Chene, 2001). Previous reports also showed that some of the p53‐interacting proteins decrease the DNA binding potential of p53 by blocking the formation of the tetramers, which provides mechanistic insights into how their presence can convert p53 target genes from an active state to a latent inactive state (van Dieck *et al.*, 2009; Foo *et al.*, 2007; Lin *et al.*, 2004; Lui *et al.*, 2015).

As part of an effort to understand the contribution of histone H4 N‐terminal tails to transcription reaction, we previously purified and characterized multiple regulatory factors that can interact with H4 tail domains. Our functional assays demonstrated that H4 tail‐associated factors have specific effects on p53‐dependent transcription reaction (Choi *et al.*, 2007). In the present study, we combine a series of biochemical and cellular techniques to further investigate roles played by these tail‐associated factors in the regulation of p53 function. Our data show that p32, one of the tail‐associated factors, participates in establishing and maintaining a repressive state of p53‐dependent transcription through its physical interaction with p53. Employing mutated forms of p32, we also demonstrate that p32 interacts with the tetramerization domain of p53 and prevents p53 from binding to its response elements. Complementary assays with cells expressing ectopic wild‐type versus p53 binding‐deficient forms of p32 confirm the physiological relevance of the observed action of p32 toward p53 inactivation through interference with p53 tetramerization. Altogether, these studies provide an unprecedented documentation of a p32 functional property directly targeting p53 transcriptional activity as well as an underlying mechanism of action both *in vitro* and *in vivo*.

## 2 Materials and methods

### 2.1 Cell lines, constructs, and antibodies

H1299 and U2OS cells were cultured in Dulbecco’s modified Eagle’s medium supplemented with 10% fetal bovine serum in an atmosphere of 5% CO_2_ at 37 °C. For mammalian expression of p32 and p53, the corresponding cDNAs were amplified by PCR and ligated into the correct reading frames of pIRESneo (Clontech Laboratories, Inc., Mountain View, CA, USA) containing 5′ FLAG or hemagglutinin (HA) coding sequences. For bacterial expression of p32, p53, SRp30c, and p66α, their cDNAs were amplified by PCR and inserted into pET15b and/or pGEX‐4T1 vectors. To generate mutant p32 expression vectors, wild‐type p32 cDNA was mutated by using Q5® Site‐Directed Mutagenesis Kit (New England Biolabs, Ipswich, MA, USA) after the construction. All constructs were verified by DNA sequencing. Further details of plasmid constructions are available upon request. Antibodies used in this study are as follows: anti‐β‐actin and anti‐FLAG antibodies from Sigma‐Aldrich, St. Louis, MO, USA; anti‐His antibody from LifeTein, Somerset, NJ, USA; anti‐lamin antibody from Active Motif, Carlsbad, CA, USA; anti‐HA antibody from Proteintech, Rosemont, IL, USA; and anti‐tubulin, anti‐p32, and anti‐p53 (DO‐1) antibodies from Santa Cruz Biotechnology, Dallas, TX, USA.

### 2.2 Preparation of recombinant proteins

Recombinant histones were expressed in *Escherichia coli* Rosetta 2 (DE3) pLysS cells (Novagen, Burlington, MA, USA) and purified as described previously (Dyer *et al.*, 2004; Mueller *et al.*, 2004). FLAG‐tagged p53, SRp30c, and p66α were expressed in *E. coli* Rosetta 2 (DE3) pLysS cells and purified with anti‐FLAG M2 agarose (Sigma‐Aldrich). His‐tagged p32 and NAP‐1 were expressed in *E. coli* Rosetta 2 (DE3) pLysS cells and purified with Ni‐NTA His·Bind Resin (Millipore, Burlington, MA, USA) according to standard protocols. FLAG‐tagged p300, ATP‐utilizing chromatin assembly factor 1 (Acf1), and ISWI were expressed in Sf21 insect cells using the Baculoviral expression system and purified with anti‐FLAG M2 agarose (An and Roeder, 2004). Glutathione *S*‐transferase (GST)‐fused proteins were expressed and purified on glutathione‐Sepharose 4B beads (GE Healthcare, Chicago, IL, USA) as described in our previous studies (Kim *et al.*, 2008).

### 2.3 *In vitro* transcription assay

Chromatin templates were assembled as described (Kim *et al.*, 2012) by using recombinant histones and ATP‐utilizing ACF chromatin assembly factor and nucleosome assembly protein 1. *In vitro* transcription assays were performed using 40 ng of pG5ML601‐280G DNA or chromatin templates for each reaction. Recombinant p32, SRp30c, and p66α were added together with p53 (20 ng) or p300 (20 ng) to transcription reactions. The radiolabeled transcripts were resolved on a 5% urea‐PAGE and detected by autoradiography (Choi *et al.*, 2007).

### 2.4 *In vitro* DNA binding assay

The biotin‐conjugated 230‐bp DNA fragments containing p53 response element (p53RE) were synthesized from p53ML plasmid by PCR amplification using a 5′‐biotinylated primer (5′‐TCTTTAAACTCGAGTGCATG‐3′) and a 3′‐primer (5′‐AGGGGGTATGGAAGGAGA‐3′) and immobilized on Dynabeads M‐280 Streptavidin (Invitrogen, Carlsbad, CA, USA). The bead‐immobilized p53RE was first incubated with FLAG‐p53 (100 ng) and p32 (80 ng) in pull‐down buffer (10 mm Tris/HCl, pH 7.5, 50 mm NaCl, 1 mm EDTA, 1 mm DTT, and 10% glycerol) with gentle shaking at 30 °C for 60 min in the presence of 20 μg polyd(I‐C) (Roche, Basel, Switzerland). The beads were then separated from the supernatant by a magnetic particle concentrator (Dynal MPC‐S). After washing three times with pull‐down buffer, equal volumes of beads were subjected to SDS/PAGE and western blot analysis.

### 2.5 RNA interference

DNA oligonucleotides encoding shRNA specific for *p32* coding region (GGATGAGGTTGGACAAGAAGA) were annealed and ligated into the lentiviral expression vector pLKO.1 (Addgene, Cambridge, MA, USA). Lentiviral particles were generated in 293T cells by transfecting plasmids encoding VSV‐G, NL‐BH, and the shRNA. For the depletion of p32 in U2OS cells, these viruses were infected and selected for 2 weeks in the presence of 2 μg·mL^−1^ puromycin. Changes in p32 expression were measured by western blotting and real‐time quantitative PCR (RT‐qPCR).

### 2.6 RT‐qPCR

Total RNA was isolated from H1299/U2OS cells using an RNeasy Mini kit (Qiagen, Hilden, Germany) and converted to first‐strand cDNA using the iScript cDNA Synthesis Kit (Bio‐Rad, Hercules, CA, USA). Real‐time RT‐PCR was carried out with QuantiTect SYBR Green RT‐PCR kit (Qiagen) according to the manufacturer’s protocol. The primers used for RT‐qPCR are listed in Table 1. Assays were normalized to *β‐actin* mRNA levels. All reactions were run in triplicate, and results were averaged.

**Table 1:** List of the primers used in RT‐qPCR.

Gene name	Forward primer (5′–3′)	Reverse primer (5′–3′)
*p21*	ATGGAACTTCGACTTTGTCAC	AGGCACAAGGGTACAAGACAGT
*NOXA*	CCAGTTGGAGGCTGAGGTTC	CGTTTCCAAGGGCACCCATG
*BAX*	CGTCCACCAAGAAGCTGAGCG	AGCACTCCCGCCACAAAGATG
*Reprimo*	CTGGCCCTGGGACAAAGAC	TCAAAACGGTGTCACGGATGT
*BTG2*	TGAGGTGTCCTACCGCATTG	GCACTTGGTTCTTGCAGGTG
*PUMA*	ACGACCTCAACGCACAGTACGA	GTAAGGGCAGGAGTCCCATGATGA
*APAF1*	GCTGCCATTTCACCAACAGT	CTCTCATTTGCTGATGTCGC
*GADD45*	AGCGGAAGAGATCCCTGTGA	CGGGAGGCAGGCAGATG
*GAPDH*	GGCCTCCAAGGAGTAAGACC	AGGGGAGATTCAGTGTGGTG
*β‐actin*	GTGGGGCGCCCCAGGCACCA	CTCCTTAATGTCACGCACGATTTC

### 2.7 ChIP

ChIP assays with H1299 cells were performed using the ChIP Assay Kit (Millipore) as recently described (Kim *et al.*, 2008). p53 (DO‐1) antibody was used for immunoprecipitation. After reversing the protein–DNA cross‐links, immunoprecipitated DNA was purified and analyzed by qPCR using the primers that amplify the p53RE regions of *p21, Noxa, BAX, Reprimo, BTG2, PUMA, APAF1*, and *GADD45* genes. The primers used for qPCR are listed in Table 2. Specificity of amplification was determined by melting curve analysis, and all samples were run in triplicate.

**Table 2:** List of the primers used in ChIP‐qPCR.

Gene name	Forward primer (5′–3′)	Reverse primer (5′–3′)
*p21*	TGGACTGGGCACTCTTGTCC	CAGAGTAACAGGCTAAGGTT
*NOXA*	GTCCAGCGTTTGCAGATG	AACGAGGTGGGAGGAGAA
*BAX*	AGATCATGAAGACAGGGGCCCTTT	TGGAGTGAGGGTGCAGAATCAGAA
*Reprimo*	GGGGAGGGGCGATAAATACC	GTAACTCCTCAGGCAGGCAA
*BTG2*	AGACGAGGCAAAGCGGTAAA	TCCAACCATTCACGGTCAGA
*PUMA*	GCGAGACTGTGGCCTTGTGT	CGTTCCAGGGTCCACAAAGT
*APAF1*	CACTGAAACATCCTCCATTA	AGGAGAATTAATGAGTTTCCAA
*GADD45*	GGATCTGTGGTAGGTGAGGGTCAGG	GGAATTAGTCACGGGAGGCAGTGCAG

### 2.8 Protein–protein interactions

For co‐immunoprecipitation assays, p53 and FLAG‐tagged wild‐type/mutant p32 proteins were co‐expressed in H1299 cells, and whole‐cell lysates were prepared from cells in cell lysis buffer (50 mm Tris/HCl, pH 7.4, 150 mm NaCl, 1 mm EDTA, 1% Triton X‐100, and protease inhibitor cocktail). The cell lysates were mixed with anti‐FLAG and anti‐p53 antibodies conjugated to beads overnight with gentle rotation at 4 °C. After removing the supernatant, the sample pellets were analyzed by western blotting with anti‐FLAG and anti‐p53 antibodies. For *in vitro* interaction studies, His‐tagged p32 and FLAG‐tagged p53 were incubated overnight with GST‐fused p53 and GST‐fused p32 proteins immobilized on glutathione‐Sepharose beads (GE Healthcare) at 4 °C in binding buffer (20 mm Tris/HCl, pH 7.3, 0.2 m KCl, 0.2 mm EDTA, 20% glycerol, 0.01% Nonidet P‐40, and protease inhibitor cocktail). After washing the beads three times with the binding buffer, bound proteins were detected by western blotting.

### 2.9 p53 tetramerization assays

For *in vitro* p53 tetramerization assays, FLAG‐tagged p53 proteins were incubated with p32 at a molar ratio of 1 : 1, 1 : 2, or 1 : 4 overnight at 4 °C in cross‐linking buffer (10 mm Tris/HCl, pH 7.5, 50 mm NaCl, 0.1 mm EDTA, 1 mm DTT, 5% glycerol, and protease inhibitor cocktail). After treating with glutaraldehyde (0.01%) for 10 min at 22 °C, the reactions were stopped by adding SDS/PAGE sample buffer, resolved on 10% SDS/PAGE, and subjected to western blotting for the detection of monomeric, dimeric, and tetrameric forms of p53. For *in vivo* p53 tetramerization assays, H1299 cells were transfected with mammalian vectors expressing wild‐type/mutant FLAG‐tagged p32 and p53. Twenty‐four hours post‐transfection, cell lysates were prepared in cross‐linking buffer and treated with glutaraldehyde at a final concentration of 0.02%. The cross‐linked lysates were subjected to western blot analysis with anti‐p53 antibody to detect p53 monomers/dimers/tetramers. To see the effects of p32 on tetramerization of endogenous p53, U2OS cells were transfected with mammalian vectors expressing wild‐type or mutant p32 for 24 h, and cell lysates were analyzed by western blotting with anti‐p53 antibody.

### 2.10 Subcellular fractionation and immunofluorescence microscopy

H1299 cells were transfected with p32/p53 expression vectors, and U2OS cells were treated with etoposide (50 µm). Cells were harvested and resuspended in cell lysis buffer (20 mm HEPES, pH 7.5, 10 mm KCl, 1 mm DTT, 1 mm PMSF, 10% glycerol, and protease inhibitor cocktail). The mixture was vortexed briefly and incubated on ice for 15 min. The nuclei were pelleted by centrifugation at 1000 ***g*** for 5 min at 4 °C, whereas the supernatant (cytoplasmic extracts) was recovered by centrifugation at 10 000 ***g*** for 20 min. Nuclei were washed with cell lysis buffer twice, resuspended in nuclear extraction buffer (20 mm HEPES, pH 7.5, 0.4 m NaCl, 1 mm DTT, 1 mm PMSF, 10% glycerol, and protease inhibitor cocktail), and incubated on ice for 30 min. The mixture was then centrifuged at 15 000 ***g*** for 10 min, and the supernatant was collected as a nuclear extract. The cytoplasmic and nuclear fractions were subjected to 12% SDS/PAGE followed by western blot analysis with anti‐FLAG (p32), anti‐HA (p53), anti‐actin, anti‐lamin, and anti‐tubulin antibodies. For immunofluorescence, cells were fixed with 4% (v/v) paraformaldehyde for 15 min, permeabilized with 0.3% Triton X‐100 for 15 min, and immunostained with anti‐FLAG (p32) and anti‐HA (p53) antibodies. Fluorescence microscopy was performed with a Zeiss microscope, and images were processed using Adobe Photoshop software. Nuclear p53 levels were quantified by assessing the nuclear area for immunofluorescence with imagej 1.48v (National Institutes of Health, Bethesda, MD, USA) software using the following formula:

$$\text{Corrected fluorescence}=\text{Total fluorescence}\;-\;\left(\text{Area selected}*\text{mean background fluorescence}\right).$$

### 2.11 Reporter gene assay

H1299 cells were plated in 12‐well plates at 50% confluence and transfected with p53RE‐luc reporter (200 ng) harboring p53REs and expression vectors for p53 (50 ng) and p32, SRp30c, or p66α (100 and 200 ng) for 24 h. Cells were harvested in Reporter Lysis Buffer (Promega, Madison, WI, USA) and assayed for luciferase activity using Odyssey System Premium (LI‐COR Bioscience, Lincoln, NE, USA).

### 2.12 Cell viability assay

Cell viability was quantified using the WST‐1 Cell Proliferation Reagent (Roche Diagnostics) according to the manufacturer’s protocol. In brief, U2OS cells (1 × 10^4^ cells·well^−1^) were treated with etoposide (50 µm) for 0, 12, 24, 48, and 72 h in 96‐well culture plates. After treatment, 10 µL of WST‐1 Cell Proliferation Reagent was added to each well followed by 2 h of incubation at 37 °C. Cell proliferation and viability were quantified by measuring absorbance at 460 nm using a microplate reader.

## 3 Results

### 3.1 p32 represses p53‐dependent transcription

**Figure 1:**
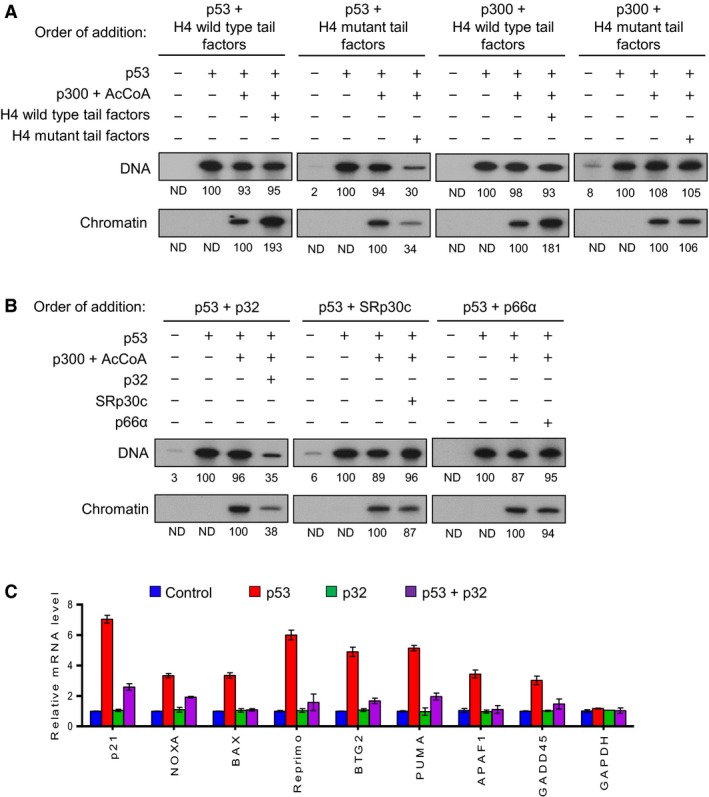
Repressive effects of p32 on p53‐dependent transcription. (A) Reconstituted nucleosome arrays and free DNA were transcribed in the presence of p53 (10 ng), p300 (20 ng), acetyl‐CoA (10 mm), and/or H4 tail‐associated factors (40 ng) as summarized in (A). The wild‐type or lysine‐mutated H4 tail‐associated factors were added together with p53 or p300 as indicated. Radiolabeled transcripts were resolved on a 5% PAGE containing 7 m urea and detected by autoradiography. Band intensity on the autoradiogram was measured by densitometry using imagej 1.48v software, and the results shown are representative of three independent experiments. (B) Transcription assays were performed under the conditions described in the legend to Fig. 1B, but recombinant p32, SRp30c, and p66α were added together with p53. (C) H1299 cells were transfected with the expression plasmids encoding p53 and p32 for 24 h. Total RNA was prepared from cells, and RT‐qPCR was performed using primers specific for the indicated p53 target genes. Primer sequences are listed in Table 1. mRNA levels from each reaction were normalized against an internal *β‐actin* control. The results shown are mean values from three independent experiments, and values derived from mock‐transfected cells are set to 1. Error bars represent SD.

Our previous study identified multiple factors that can specifically associate with wild‐type or lysine‐mutated N‐terminal tail domains of histone H4 (Choi *et al.*, 2007) (Fig. S1). In transcription experiments using these associated factors, we noticed that wild‐type H4 tail‐associated factors, but not mutant tail‐associated factors, can modulate p53‐dependent chromatin transcription when added together with p300 to reactions (Choi *et al.*, 2007). To check whether these tail‐associated factors can affect transcription more precisely, we first carried out the order‐of‐addition experiments as summarized in Fig. S2A. In our assay system using a plasmid containing p53RE, chromatin transcription is dependent upon activator p53, cofactor p300, and acetyl‐CoA, while histone‐free DNA templates can get transcribed solely in the presence of p53 (Fig. 1A). Consistent with our published data (Choi *et al.*, 2007), simultaneous addition of wild‐type H4 tail‐associated factors and p300 boosted p53‐dependent transcription from chromatin templates but not from DNA templates (Fig. 1A). The replacement of H4 wild‐type tail‐associated factors with mutant tail‐associated factors in this reaction generated no changes in transcription reactions. When the effects of wild‐type tail‐associated factors were examined by adding them together with p53, transcriptional activation from chromatin templates was still observed. Interestingly, however, parallel transcription assays in which the mutant tail‐associated factors were added together with p53 resulted in a distinct repression in both DNA and chromatin transcription (Fig. 1A).

Since p32, SRp30c, and p66α were specifically associated with H4 mutant tails (Fig. S1), we wished to define which of these factors are directly involved in the observed repression. For this purpose, p32, SRp30c, and p66α were expressed in bacteria and affinity‐purified as described in section 2.22.2 and shown in Fig. S2B. It is worth noting that p32 is synthesized as a pro‐protein of 282 amino acids residing in the cytoplasm before undergoing cleavage of amino acids 1–73 to generate the mature form of the protein (Murakami *et al.*, 1993). When transcription assays were performed with each of these recombinant factors, little or no repression in transcription was observed by the addition of SRp30c or p66α together with p53 (Fig. 1B). However, the addition of mature p32 protein together with p53 resulted in a dramatically lower level of transcription, strongly supporting that p32 is mainly responsible for the repressive effects of the mutant tail‐associated factors (Fig. 1B). As expected, p32 exerted no measurable effects on p53 transactivation when it was added to transcription reactions together with p300 (not shown). To further confirm our finding, we also conducted quantitative RT‐PCR (RT‐qPCR) analysis of eight representative p53 target genes using the p53‐null H1299 lung cancer cell line. As summarized in Fig. 1C, the expression of ectopic p53 activated transcription of p53 target genes, but co‐expression of p32 significantly repressed the transactivation of p53 target genes in the cells. In all cases, the mRNA levels of the non‐p53 target gene *GAPDH* remain unchanged. In checking the possible effects of SRp30c and p66α, they failed to diminish the extent of p53 target gene expression in p53‐transfected cells (Fig. S3). Also, these results are in complete agreement with data indicating that co‐expression of p53 with p32 led to much lower activity of p53‐responsive luciferase reporter gene compared to its transfection with SRp30c and p66α (Fig. S4). Taken together with our *in vitro* data, these experiments unequivocally demonstrate a selective inhibitory action of p32 against p53‐dependent transcriptional activation.

### 3.2 p32 binds to p53 tetramerization domain

**Figure 2:**
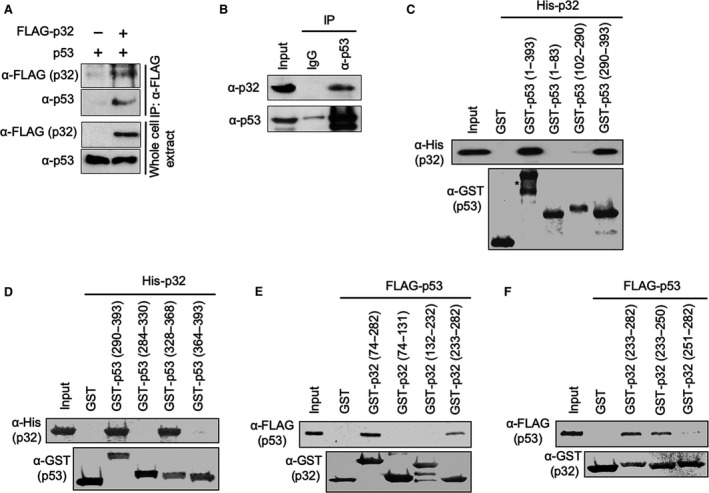
Direct interaction of p32 with p53. (A) H1299 cells were transfected with FLAG‐tagged p32 and/or p53 for 24 h. Extracts were prepared in lysis buffer and immunoprecipitated with anti‐FLAG antibody. Western blot analysis was performed with p53 antibody to detect the presence of p53. (B) Whole‐cell lysates were prepared from U2OS cells, immunoprecipitated with anti‐p53 antibody or control IgG, and analyzed by western blotting with anti‐p32 and anti‐p53 antibodies. Input represents 10% of the cell extracts used in immunoprecipitation. (C) GST alone or the indicated GST‐p53 fusions were immobilized on glutathione‐Sepharose beads and incubated with His‐tagged p32. After extensive washing, bound p32 proteins were fractionated by 12% SDS/PAGE and examined by western blotting with anti‐His antibody. Ten percent of the input proteins also shown. The right panel shows the schematic illustration of p53 and its deletion mutants used in the assays. Numbers indicate amino acid residues. (D) *In vitro* binding assays with His‐tagged p32 were performed essentially as described in (C), but p53 C‐terminal subdomains were used as indicated. (E) FLAG‐tagged p53 was incubated with immobilized GST or indicated GST‐p32 fusions, and bound p53 proteins were visualized by western blotting with anti‐FLAG antibody. Input represents 10% of p32 protein used in the binding reactions. (F) *In vitro* binding assays with FLAG‐tagged p53 were performed essentially as described in (E), but indicated p32 C‐terminal subdomains were used.

Overall, the transcription data presented above establish the role for p32 as a negative regulator of p53‐mediated transactivation. However, it is not clear whether the repressive effects of p32 reflect its physical interaction with p53. To check this possibility, we transfected H1299 cells with plasmids expressing p53 and FLAG‐tagged p32 and immunoprecipitated whole‐cell lysates with anti‐FLAG antibody. In addition to p32, we could confirm the presence of p53 in our immunoprecipitates by western blot analysis (Fig. 2A). These binding data were further supported by our finding that p53 antibody is able to co‐immunoprecipitate endogenous p32 from lysates of human osteosarcoma‐derived U2OS cells bearing wild‐type p53 (Fig. 2B). To determine more precisely the nature of the observed interaction, we then conducted *in vitro* pull‐down assays using bacterially produced His‐tagged p32 and GST‐fused p53. As shown in Fig. 2C, GST‐p53 efficiently interacted with His‐p32, whereas GST alone did not. In mapping p32‐interacting region of p53, the binding of the C‐terminal region (residues 290–393) was readily detectable, but the N‐terminal (residues 1–83) and central (residues 102–290) regions showed no interaction with p32. In similar binding experiments with truncated versions of p53 C‐terminal region, p32 interacted with residues 328–368 of p53, but not with the remainders (residues 284–330 and 364–393) of p53 C‐terminal region (Fig. 2D). These results reinforce the conclusion that the primary p32‐binding capacity of p53 resides in the tetramerization domain (residues 328–368). In parallel binding experiments with GST‐p32 and FLAG‐tagged p53, the C‐terminal domain (residues 233–282) of p32 retained strong affinity for p53, while no apparent interaction was observed with N‐terminal (residues 74–131) and central (residues 132–232) domains of p32 (Fig. 2E). When two subdomains of p32 were used in our binding assays, p53 efficiently binds to residues 233–250 but not residues 251–282 of p32 (Fig. 2F), indicating that a small subdomain in the C‐terminal domain of p53 plays a major role in p32 binding to p53.

**Figure 3:**
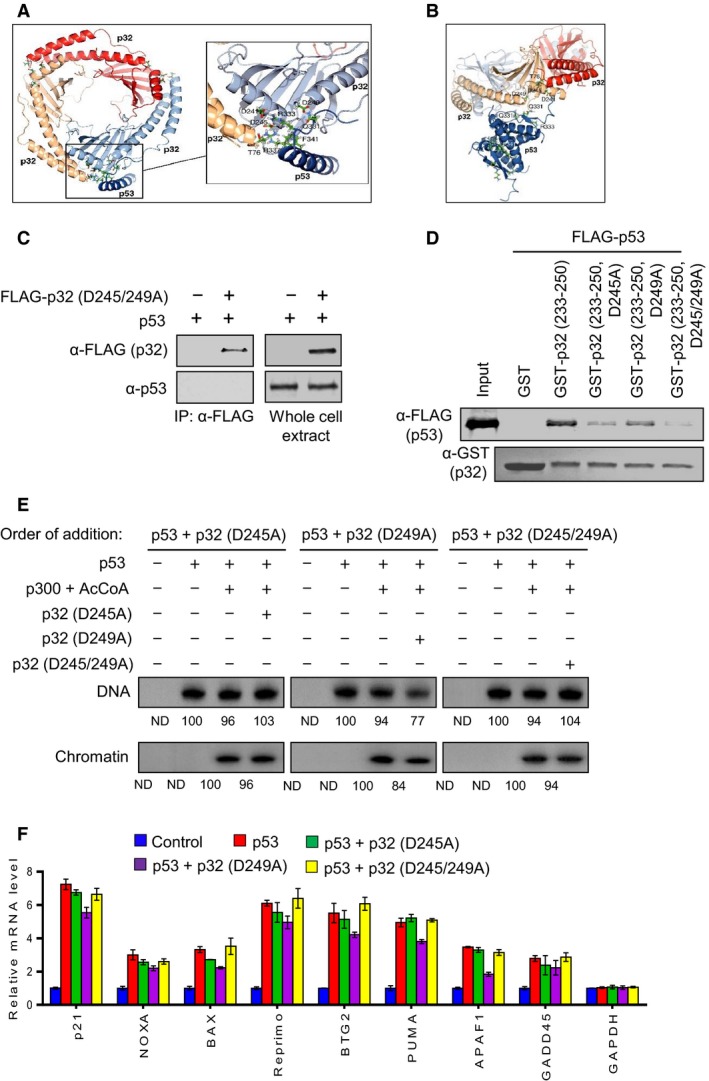
Effects of p32 mutations on p32–p53 interaction. (A) Model of trimeric p32–monomeric p53 interaction. Docking predictions favor p53(Gln331)–p32(Asp249), p53(Arg333)–p32(Asp241), and p53(Arg337)–p32(Asp245) contacts and hydrophobic interactions involving p53(Phe341). (B) Model of trimeric p32–tetrameric p53 interaction. p32 was predicted to engage tetrameric p53 only via p53(Gln331)–p32(Asp249). Protein Data Bank entries 3sak (p53), 1aie (p53), and 1p32 (p32) were used in docking simulations using the program cluspro 2.0 (Kozakov et al., 2017; Mittl et al., 1998). The structure of p32‐bound monomeric p53 was assumed to resemble its structure in the tetrameric p53 assembly. (C) Whole‐cell extracts were prepared from H1299 cells expressing p53 and FLAG‐tagged D245/249A‐mutated p32 and immunoprecipitated with anti‐FLAG antibody. Western blot analysis was performed with p53 antibody to detect the presence of p53 in the precipitated proteins. (D) GST pull‐down assays were conducted as in Fig. 2F, but using GST‐p32 (233–250) bearing point mutations at D245 and/or D249. (E) *In vitro* transcription assays were conducted as described in Fig. 1C, except that p32 proteins carrying the indicated mutations were used. (F) H1299 cells were transfected with the expression plasmids encoding p53 and p32 mutants for 24 h as indicated. Total RNA was prepared from cells, and RT‐qPCR was performed using primers specific for the indicated genes. mRNA levels from each reaction were normalized against an internal *β‐actin* control. The results shown are mean values from three independent experiments, and values derived from mock‐transfected cells are set to 1. Error bars represent SD.

To identify amino acid residues critical for the above‐outlined interaction interface, we next created a structural model of the p32–p53 complex using crystal structures of p32 and p53. With monomeric p53, interactions near the apex of the p32 helices were indicated in our structural model (Fig. 3A). Specifically, electrostatic p53(Gln331)–p32(Asp249), p53(Arg333)–p32(Asp241), and p53(Arg337)–p32(Asp245) contacts and hydrophobic interactions involving p53(Phe341) were apparent. In tetrameric p53, p53(Gln331) and p53(Arg337) engage p53(Asp352) across constituent monomers. Likewise, p53(Phe341) is buried in the tetramerization interface and inaccessible. Only p53(Gln331)–p32(Asp249) interactions still appear possible according to our model (Fig. 3B). Thus, we propose p32 to destabilize the p53 tetramer by engaging residues that form the tetramerization interface. Consistent with this view, we were unable to detect p53 proteins in p32 immunoprecipitates from extracts of H1299 cells expressing wild‐type p53 and D245/D249‐mutated p32 (Fig. 3C). Based on our model, we expected p32 (D245A) to significantly interfere with the p32–p53 interactions and p32(D249A) to do so moderately. *In vitro* binding assays employing GST‐p32 D245A and D249A mutant proteins demonstrated that the interaction of p32 with p53 was inhibited strongly by D245A mutation and weakly by D249A mutation (Fig. 3D). We also found that an almost complete abrogation of the p32–p53 interaction requires the alanine substitution of both D245 and D249 (Fig. 3D). Given that p32 function in attenuating p53 transactivation involves its direct interaction with p53, we also investigated the impact of mutating D245 and D249 on p32 transrepressive activity. For this purpose, p32, p32 (D245A), p32 (D249A), and p32 (D245/249A) were expressed in bacteria and affinity‐purified as described in section 2.22.2 and shown in Fig. S2C. As shown in Fig. 3E, the repressive effect of p32 on p53 transactivation was rescued by individual mutation of D245A and D249A of p32. Similarly, the addition of D245/D249‐mutated p32 to the reactions generated no changes in p53‐dependent transcription. Consistent with these *in vitro* data, parallel transfection experiments showed that the repressive effects of p32 on p53 transactivation were rescued by mutation of D245A and/or D249A of p32 (Fig. 3F). These data overall indicate the p32–p53 interaction to be centered near the apex of p32 α‐helices, supporting p32‐mediated collapse of p53 tetramerization and verifying that p32 directly modulates p53 transactivation.

### 3.3 p32 inhibits p53 DNA binding and tetramerization

**Figure 4:**
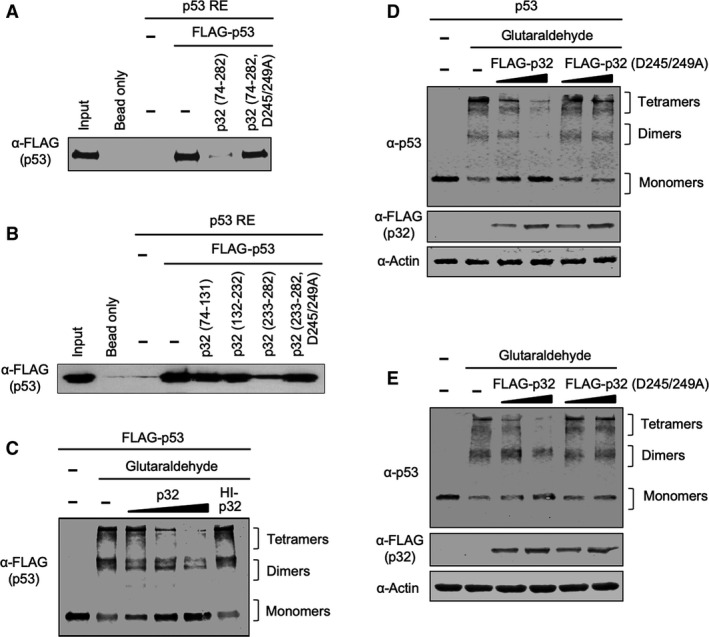
Inhibition of p53 DNA binding and tetramerization by p32. (A) A biotinylated DNA fragment containing RE was synthesized and immobilized on streptavidin‐coated magnetic beads. FLAG‐tagged p53 was incubated with the DNA fragment linked to the beads in the presence or absence of His‐tagged p32 wild‐type and mutant (D245/249A). After several washing steps, the bound fraction was subjected to SDS/PAGE and detected by western blotting. (B) *In vitro* binding assays with immobilized p53 RE were performed essentially as in (A), but p32 deletion mutants and C‐terminal domain carrying D245/249A mutations were used as indicated. (C) FLAG‐tagged p53 proteins were treated with 0.01% glutaraldehyde in the presence or absence of p32 at 22 °C for 10 min. The molar ratio of p53 and p32 was 1 : 1, 1 : 2, or 1 : 4 as indicated. Heat‐inactivated (HI) p32 was used at a p53/p32 molar ratio of 1 : 4 in control reactions. The samples were resolved on 10% SDS/PAGE and analyzed by western blotting with anti‐FLAG antibody. The positions of the monomers, dimers, and tetramers are indicated on the right. (D) H1299 cells were transfected with plasmids coding p53 and wild‐type/mutant p32 for 24 h as indicated at the top of the figure. After preparing cell lysates, aliquots (10 μg) of protein extracts were treated with 0.02% glutaraldehyde for 10 min at 22 °C. The samples were resolved on 10% SDS/PAGE and analyzed by western blotting with anti‐p53 and anti‐FLAG antibodies. (E) Tetramerization potential of wild‐type or mutant p32 was assessed as in (D), but using cell lysates (40 μg) from U2OS cells transfected with wild‐type or mutant p32 for 24 h.

On the basis of the fact that p32‐bound p53 proteins are defective in both DNA and chromatin transcription, we postulated that these functional defects are attributable to alterations in the DNA binding capacity of p53. To check this possibility, the 5′‐biotinylated DNA fragments containing p53RE were PCR‐amplified and immobilized on streptavidin‐coated paramagnetic beads for binding assays (Fig. S5). The bead‐immobilized p53RE DNA was incubated with FLAG‐tagged p53 in the presence of wild‐type or mutant p32 proteins under the conditions used in the transcription assays. The immobilized DNA was separated from the reactions, washed extensively with binding buffer to remove unbound proteins, and analyzed by western blotting with anti‐FLAG antibody. Expectedly, p53 binding to the immobilized p53RE DNA was readily detectable in the absence of p32 (Fig. 4A). However, the observed p53 binding was lost almost completely when p32 wild‐type was added to binding reactions. The inhibitory effects of p32 were due to its direct binding to p53, because we could still observe a stable binding of p53 to its RE DNA in the presence of p53 binding‐deficient p32 mutant with substitutions at D245 and D249 (Fig. 4A). Additionally, in checking the effects of specific regions of p32 under the same conditions, we found that the C‐terminal domain (amino acids 233–282) of p32 is sufficient to inhibit the binding of p53 protein to its response elements (Fig. 4B)—indicative of C‐terminal domain‐dependent function of p32.

We next attempted to understand how p32 interferes with the transactive function of p53 at target genes. Because p32 binds directly to the tetramerization domain of p53, a potential mechanism of p32 action is that p32 binding to p53 modulates the tetramerization capacity of p53. For the purpose of checking this possibility, p53 proteins were incubated with p32 in the presence or absence of glutaraldehyde, and cross‐linked products were analyzed by western blotting. Cross‐linking of p53 proteins resulted in two distinct cross‐linked forms with sizes corresponding to those of the p53 dimer and tetramer (Fig. 4C). Our analysis also revealed that the addition of increasing quantities of p32 markedly diminishes the ability of p53 to form tetramers in the reaction. The observed effects were specific, since heat‐inactivated p32 failed to show any inhibitory activity toward p53 tetramerization (Fig. 4C). In order to investigate whether p32 has similar effects on cellular p53 proteins, H1299 cells were cotransfected with p53 and p32 wild‐type or interaction‐defective D245/249A mutant and cross‐linked with glutaraldehyde. Western blotting of cross‐linked lysates clearly indicated that co‐expression of p32 wild‐type abrogates p53 tetramerization in H1299 cells (Fig. 4D). On the contrary, no changes in p53 tetramerization were observed in the cells expressing p53 and p32 D245/249A mutant (Fig. 4D). Likewise, when p53‐positive U2OS cells were transfected with p32 wild‐type, endogenous p53 was unable to form dimers and tetramers (Fig. 4E). An analogous experiment also showed that overexpression of p53‐interaction‐defective p32 mutant did not have any impact on the tetramerization capacity of p53 (Fig. 4E), strongly supporting that the blockade of p53 tetramerization is a critical component of p32‐induced p53 suppression.

### 3.4 p32 restricts p53 occupancy at target genes

**Figure 5:**
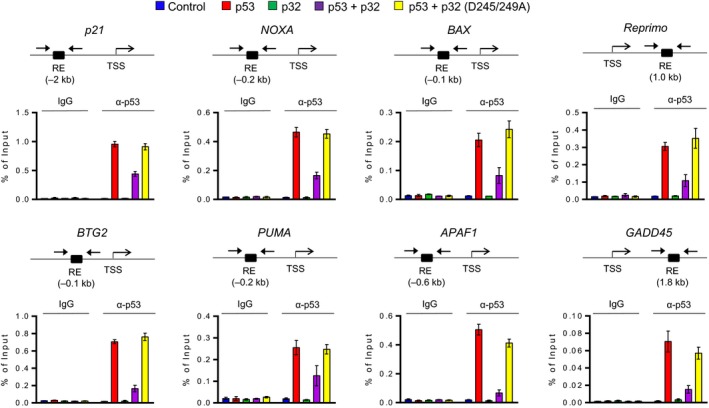
Increased recruitment and function of p53 after p32 knockdown. Chromatin was prepared from control H1299 cells and H1299 cells expressing p53 and/or p32 and analyzed by ChIP assays using anti‐p53 antibody. Precipitated DNA was amplified with primers depicted at the top and listed in Table 2. Percent input is determined as the amount of immunoprecipitated DNA relative to input DNA. Error bars represent the SD obtained from three independent experiments.

Our finding that p32 interacts and interferes with p53 tetramerization suggests that the observed effects may be directly linked to p32‐induced inhibition of p53 binding to target genes. Therefore, we conducted ChIP assays to measure the effects of p32 expression on the levels of p53 occupancy at target genes. Cross‐linked chromatin was isolated from control H1299 cells and H1299 cells expressing p53 and/or p32 and sonicated to mono‐ and dinucleosomes after cross‐linking. The precipitated nucleosomal DNA was extracted and amplified by qPCR using primers specific for p53RE regions of target genes. Although the precipitation efficiency was slightly different among the target genes, we were able to detect p53 ChIP signals in the vicinity of response elements in p53‐transfected cells (Fig. 5), indicating that the ectopic p53 stably binds to p53RE in cells. Under these assay conditions, we observed a severe reduction in p53 levels at target genes in response to p32 expression, confirming the involvement of p32 in regulating the extent of target gene occupancy by p53. When ChIP experiments were performed after the expression of p53‐interaction‐defective p32 D245/249A mutant, a distinct increase in p53 localization around response elements was also apparent (Fig. 5). These results exclude the possibility that the observed effects of p32 are generated by antibody cross‐reactivity with off‐target proteins, and indicate that p53‐targeted function of p32 is indeed accurately determined in our assays. Together, our ChIP‐qPCR data support the notion that the repressive activity of p32 toward p53 transactivation is generated from its restrictive effects on p53 occupancy of target genes.

### 3.5 p32 promotes p53 cytoplasmic localization by nuclear export

**Figure 6:**
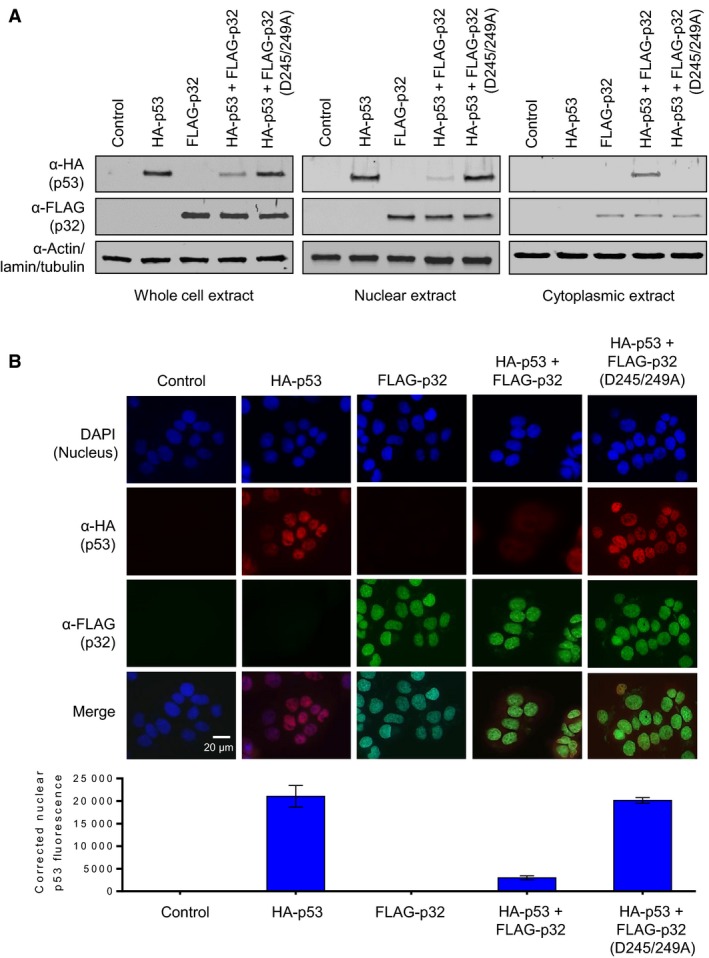
Stimulation of p53 nuclear export by p32. (A) H1299 cells were transfected with expression vectors for wild‐type and mutant FLAG‐p32 and HA‐p53 proteins, as indicated. Whole‐cell extracts, cytoplasmic extracts, and nuclear extracts were prepared and analyzed by western blotting with anti‐FLAG and anti‐HA antibodies. The blot presented is representative of three independent experiments. (B) H1299 cells were transfected with FLAG‐p32 and HA‐p53 expression vectors and immunostained with anti‐FLAG and anti‐HA antibodies. For each experiment, 20 cells were examined. The representative immunostaining images are shown. Scale bar, 20 μm. Adjoining graph represents the amount of nuclear p53 fluorescence in each group quantified by imagej 1.48v software. Error bars represent SD.

The tetramerization domain of p53 contains a leucine‐rich NES that directs p53 from the nucleus to the cytoplasm (Stommel *et al.*, 1999). When p53 is in tetrameric form, this NES remains hidden for the nuclear function of p53. If p53 tetramerization is inhibited, NES is exposed to allow export of p53 from the nucleus to the cytoplasm for ubiquitin‐dependent proteasomal degradation (Kamada *et al.*, 2016; Lee *et al.*, 1994). Thus, intrigued by the negative impact of p32 on p53 tetramerization, we next sought to evaluate whether p32 has a role in regulating p53 nuclear export. To this end, wild‐type/mutant mature p32 and p53 were expressed in H1299 cells for 24 h, and the levels of p32 and p53 in the cytoplasmic versus nuclear fractions were analyzed by western blotting. The quality of cytoplasmic and nuclear fractionations was determined by the presence of cytoplasmic tubulin and nuclear lamin in the fractions. When individually expressed in H1299 cells, p53 and mature p32 proteins were predominantly located in the nucleus and rarely detected in the cytoplasm (Fig. 6A). The results in Fig. 6A also indicate that co‐expression of p53 with p32 leads to a significant decrease in nuclear levels of p53 protein. In checking the levels of p53 protein in the cytoplasm of these cells, we detected low levels of p53, presumably because cytoplasmic p53 proteins were quickly subjected to proteasome‐mediated degradation. Since total cellular levels of p32 protein were not affected by p53 expression (Fig. 6A), the p32–p53 interaction acts in only one direction and results in cytoplasmic translocation and degradation of p53. In parallel experiments in which p53 levels were analyzed in cells transfected with p53‐interaction‐defective p32 D245/249A mutant, no changes in the level of nuclear p53 protein were observed.

To further examine the role of p32 in p53 nucleus‐to‐cytoplasm translocation, we also performed immunofluorescence analysis. Figure 6B shows representative examples of immunostained cells, as well as the ratio between cells with nuclear and cytoplasmic staining of p32 and p53. HA‐p53 alone displayed predominantly a nuclear localization pattern. Also, high levels of FLAG‐p32 were distributed throughout the nucleus with some traces in the cytoplasm. When cells were cotransfected with combination of HA‐p53 and FLAG‐p32, a significant reduction in the number of cells with nuclear p53 was observed (Fig. 6B). Again, a minor staining of FLAG‐p53 in the cytoplasm of p32‐transfected cells suggests that p53 is subjected to degradation immediately following its export to the cytoplasm. Expectedly, the intensity of nuclear p53 staining was not significantly decreased after co‐expression of p53‐interaction‐defective p32 D245/249A mutant. Together, these data provide insights into the specific regulatory mechanisms whereby p32 influences the tumor suppressor function of p53 by promoting its nuclear export and degradation.

### 3.6 p32 impairs p53 transactivation in response to DNA damage

**Figure 7:**
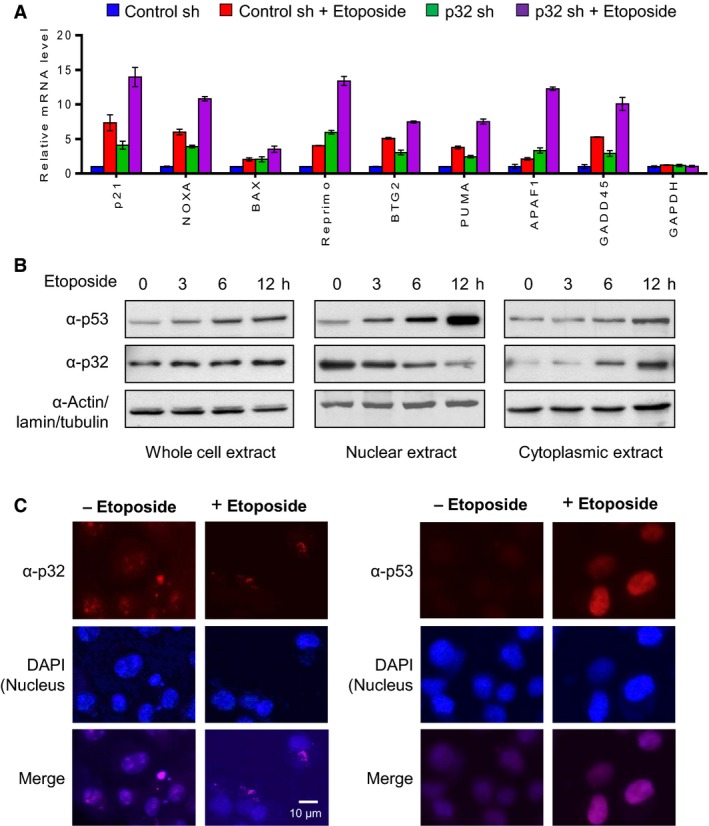
Negative regulation of p53 by p32 in response to DNA damage. (A) Control and p32‐depleted U2OS cells were mock‐treated or treated with 50 µm etoposide for 12 h. RNA samples were prepared and analyzed by RT‐qPCR using primers specific for eight p53 target genes and *GAPDH* control gene. The values are expressed as fold changes from the mRNA levels in undepleted control cells. Data represent the means ± SD of three independent experiments. (B) U2OS cells were treated with etoposide (50 µm) for 0, 3, 6, and 12 h, and whole‐cell extracts, cytoplasmic extracts, and nuclear extracts were prepared and analyzed by western blot with anti‐p53 and anti‐p32 antibodies. The blot presented is representative of three independent experiments. (C) After treating with etoposide as in (A), U2OS cells were immunostained with anti‐p32 and anti‐p53 antibodies. For each experiment, 100 cells were examined. The representative immunostaining images are shown. Scale bar, 10 μm.

As p32 interferes with p53 tetramerization and enhances p53 translocation to the cytoplasm, we next wanted to know whether the observed effects are directly linked to p32‐induced suppression of p53 transactivation in response to DNA damage. To this end, we prepared p32‐depleted U2OS cells by using a lentiviral vector system. Western blotting and RT‐qPCR analyses confirmed that our infection of U2OS cells with lentiviral vectors expressing p32 shRNA potently ablated the expression of p32 in the cell (Fig. S6A). More importantly, it was apparent from a comparison of p53 target gene expression in mock‐depleted control and p32‐depleted cells that p32 knockdown led to increased expression of p53‐responsive genes (Fig. 7A). Since etoposide induces p53 transactivation and apoptosis from DNA damage, we also treated U2OS cells with 50 μm etoposide for 12 h and analyzed the effects of p32 on the expression of p53 and its target genes. As summarized in Fig. 7A, etoposide‐treated cells showed much higher levels of p53 target gene expression when p32 is depleted, indicating the negative role played by p32 in p53 transactivation (Fig. 7A). These results exclude the possibility that the observed effects of p32 shRNA are generated by off‐target activity, and indicate that transrepressive function of p32 is indeed accurately established in our assays. Consistent with these observations, the viability of etoposide‐treated U2OS cells also decreased significantly following stable knockdown of p32 (Fig. S6B).

For the purpose of evaluating a possible role of p32 in regulating p53 nuclear export upon DNA damage, we next treated U2OS cells with etoposide for 0, 3, 6, or 12 h and analyzed the levels of p32 and p53 in the cytoplasmic versus nuclear fractions at each time point by western blotting. As shown in Fig. 7B, etoposide treatment of U2OS cells led to a progressive decrease in nuclear p32 levels and increase in a cytoplasmic accumulation of p32. Since etoposide‐induced DNA damage only modestly increased total cellular levels of p32 protein, cytoplasmic translocation of p32 may be a critical event for attenuating the repressive action of p32 against p53‐mediated transactivation. In parallel experiments in which changes in p53 levels were analyzed over the same time periods, a rapid increase in the level of p53 in the nucleus was evident after etoposide treatment. Low levels of p53 were found to reside in the cytoplasm over the time periods, presumably because cytoplasmic p53 proteins were quickly subjected to proteasome‐mediated degradation. Consistent with these observations, our immunofluorescence staining revealed that treating U2OS cells with etoposide for 12 h significantly decreased the intensity of nuclear p32 (Fig. 7C, left panel). Furthermore, the levels of p53 were inversely proportional to those of p32 in U2OS cell nuclei after etoposide‐induced DNA damage (Fig. 7C, right panel). Together, these data provide insights into the specific regulatory mechanisms whereby p32 influences the tumor suppressor function of p53 by promoting its nuclear export and degradation.

## 4 Discussion

The p53 transcriptional program is a central theme in mediating cellular response to a wide range of environmental stresses. Deregulation of p53 signaling pathway has been associated with the pathogenesis of cancer and other diseases. Though much efforts over the past two decades have focused on the direct action of p53 during its target gene transcription, increasing evidence also suggests that nuclear exclusion and degradation of p53 play important roles in the maintenance of a balanced p53 signaling. In the present study, we used defined *in vitro* assay systems and provided new evidence documenting that p32 is a negative regulator of p53‐dependent transcription from both DNA and chromatin templates. Importantly, our order‐of‐addition experiments confirmed that p32 should be added to transcription reactions together with p53 for its repressive action, indicative of a specific functional role of p32 targeting DNA‐unbound free state of p53. Another argument in favor of p32 to act as a master p53 repressor comes from our cellular experiments showing that the expression of p32 in p53‐transfected H1299 cells leads to a reduced transcription of p53 target genes. In further support of these results, p32 was able to constitute a repressive barrier to the transcriptional activity of p53 in both undamaged and damaged p53‐positive U2OS cells. The ability of p32 to mediate the repression of p53‐dependent transactivation depends on its direct association with p53, as we demonstrated by a series of biochemical and cellular experiments. In addition, our mutagenesis studies based on the structural modeling of p32–p53 complex revealed that D245 and to a lesser extent D249 are crucial for p32 in blocking the formation of p53 tetramers. Consequently, we were able to show that these mutations are sufficient to abolish the roles played by p32 in inhibiting the transcriptional potential of p53 toward target genes. This is the first study indicating that p32 is capable of establishing an inactive state of DNA repair and apoptotic genes and does so in a p53‐dependent manner.

In an effort to decipher the molecular basis underlying p32 transrepressive activity, we found that p32 directly interacts with the C‐terminal tetramerization domain of p53 and successfully competes for p53 residues that mediate its tetramerization. p53 binds its DNA response elements most efficiently as a tetramer, and tetrameric p53 is most effective for transactivation of target genes. Thus, the defective tetramerization property of p32‐bound p53 in our cross‐linking assays also fits well to the idea that the interaction of p32 with p53 tetramerization domain blocks the ability of p53 to form tetramer and negatively regulates p53 function in activating the expression of DNA repair and apoptotic genes. p32 is among a few proteins that disfavor p53 tetramerization (Clore *et al.*, 1995) and, to our knowledge, we provide the first model for the structural basis of this mechanism. Another interesting result obtained from our study is that p32‐mediated inhibition of p53 tetramerization exposes a NES embedded within the tetramerization domain and triggers the nuclear export of p53 for cytoplasmic degradation. We further demonstrated that etoposide‐induced DNA damage generated high levels of p53 expression, relocated p32 to the cytoplasm, stimulated p53 to tetramerize, and restored p53 transcriptional activity in the nucleus. This finding is of particular importance because it indicates the use of a unique mechanism for linking p32 nuclear function to p53 inactivation through inhibition of tetramer formation which opens the NES and allows access to the nuclear export machinery. Since the expression levels of p32 have been found higher in cancer cells with respect to those in normal cells (Chen *et al.*, 2009; Rubinstein *et al.*, 2004), our results are of significance in relation to the key role of p32 in disabling p53 function during cancer development. Therefore, the new level of insight we provide into p53‐targeted p32 function promises to be useful for understanding p32‐driven oncogenesis as a potential new target for cancer therapy in the future.

## 5 Conclusions

In conclusion, we investigated the possible effects of p32 on p53 transactivation using defined experimental systems. Our initial characterization demonstrated that p32 plays a critical role in inactivating p53‐dependent transcription. We extended these findings by showing that the C‐terminal tetramerization domain of p53 is the target for the observed inhibitory function of p32. Mechanistically, the negative effects of p32 on p53 transactivation result from the abrogation of p53 tetramer formation as well as the nuclear export of p53 to the cytoplasm for degradation. Thus, our findings argue that the direct interaction of p32 with p53 has a critical function in antagonizing the p53 tumor suppressor pathway. It remains a challenge to understand how p32 cooperates with other factors that have been reported to participate in p53‐induced cell growth arrest, apoptosis, and DNA repair.

## Conflict of interest

The authors declare no conflict of interest.

## Author contributions

NG, JK, and WA conceived and designed the study. AS and TU generated a structural model of the p32–p53 complex. NG, JK, and YS performed the experiments with contributions of TU and WA. NG and WA analyzed the data and wrote the manuscript. All authors read and approved the final manuscript.

## Supporting information


**Fig. S1** **.** List of the factors interacting with wild‐type (wt) and mutant (mt) H4 N‐terminal tails.

**Fig. S2** **.** (A) Schematic representation of the *in vitro* transcription assay. (B, C) Western blotting of bacterial purified recombinant proteins.

**Fig. S3** **.** Effects of SRp30c and p66α on p53‐target gene expression.

**Fig. S4** **.** p32 interferes with p53 transcriptional activity.

**Fig. S5** **.** Schematic summary of the *in vitro* DNA binding assay, related to Figs 4A,B.

**Fig. S6** **.** (A) p32 depletion in U2OS cells. (B) Effect of etoposide on control and p32‐depleted U2OS cells.
