# Supplementary figures and images for: p32 is a negative regulator of p53 tetramerization and transactivation
Source: Mol Oncol. 2019 Jul 30;13(9):1976–92. doi: 10.1002/1878-0261.12543 (PMC6717765; doi:10.1002/1878-0261.12543)

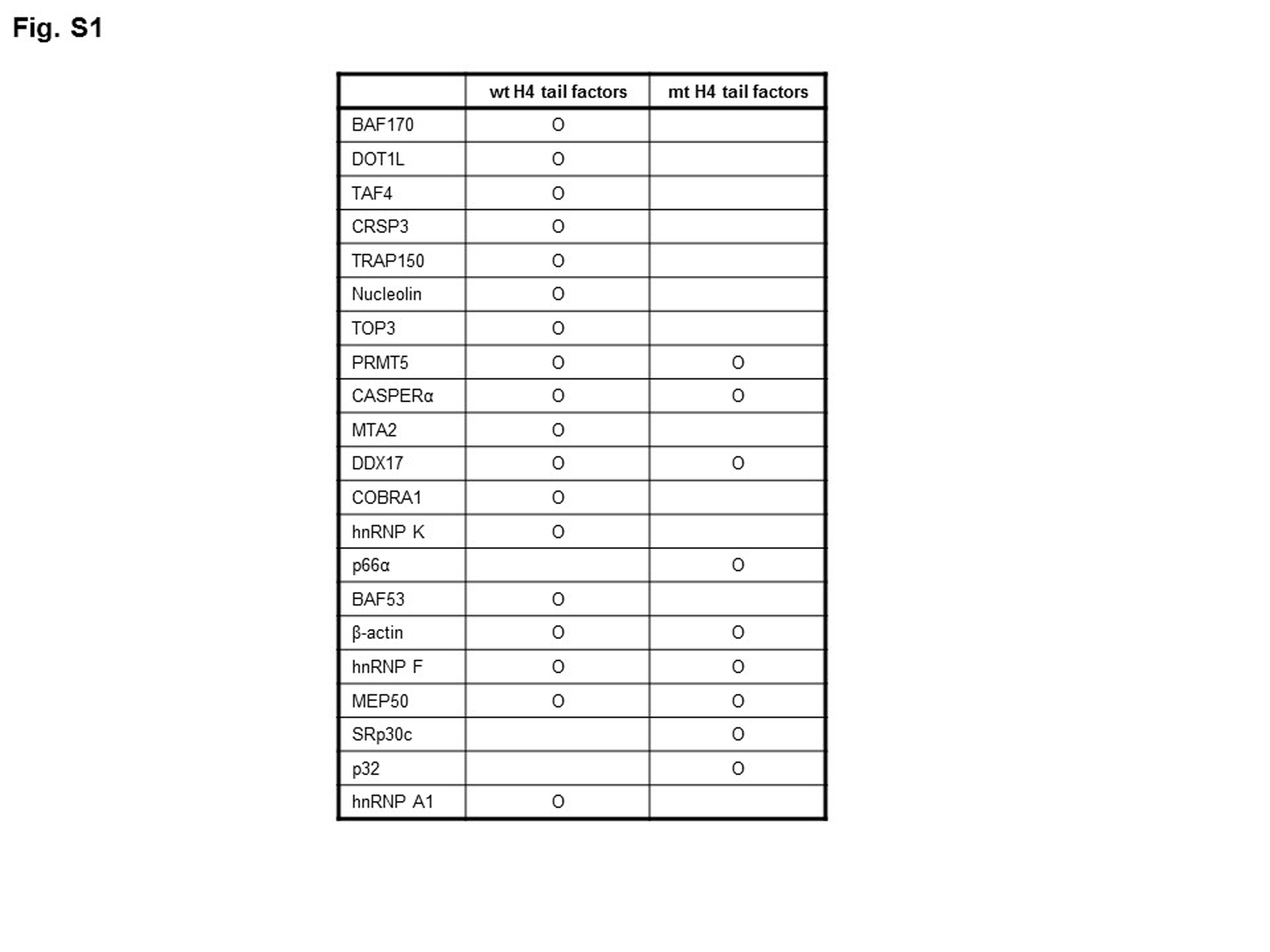

Supplement: Supplementary file 1 — Fig. S1 . List of the factors interacting with wild‐type (wt) and mutant (mt) H4 N‐terminal tails. [file MOL2-13-1976-s001.JPG]

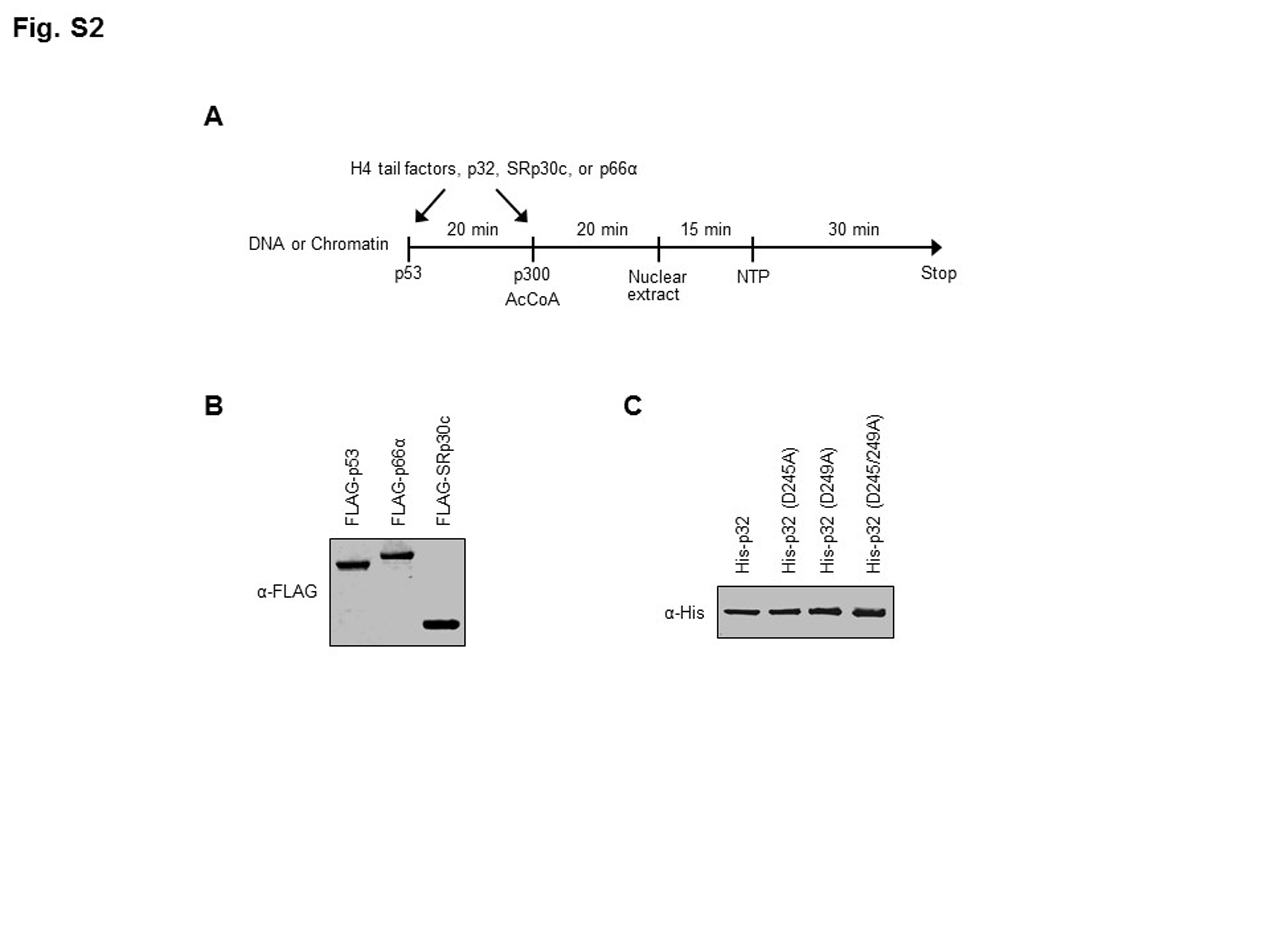

Supplement: Supplementary file 2 — Fig. S2 . (A) Schematic representation of the in vitro transcription assay. (B, C) Western blotting of bacterial purified recombinant proteins. [file MOL2-13-1976-s002.JPG]

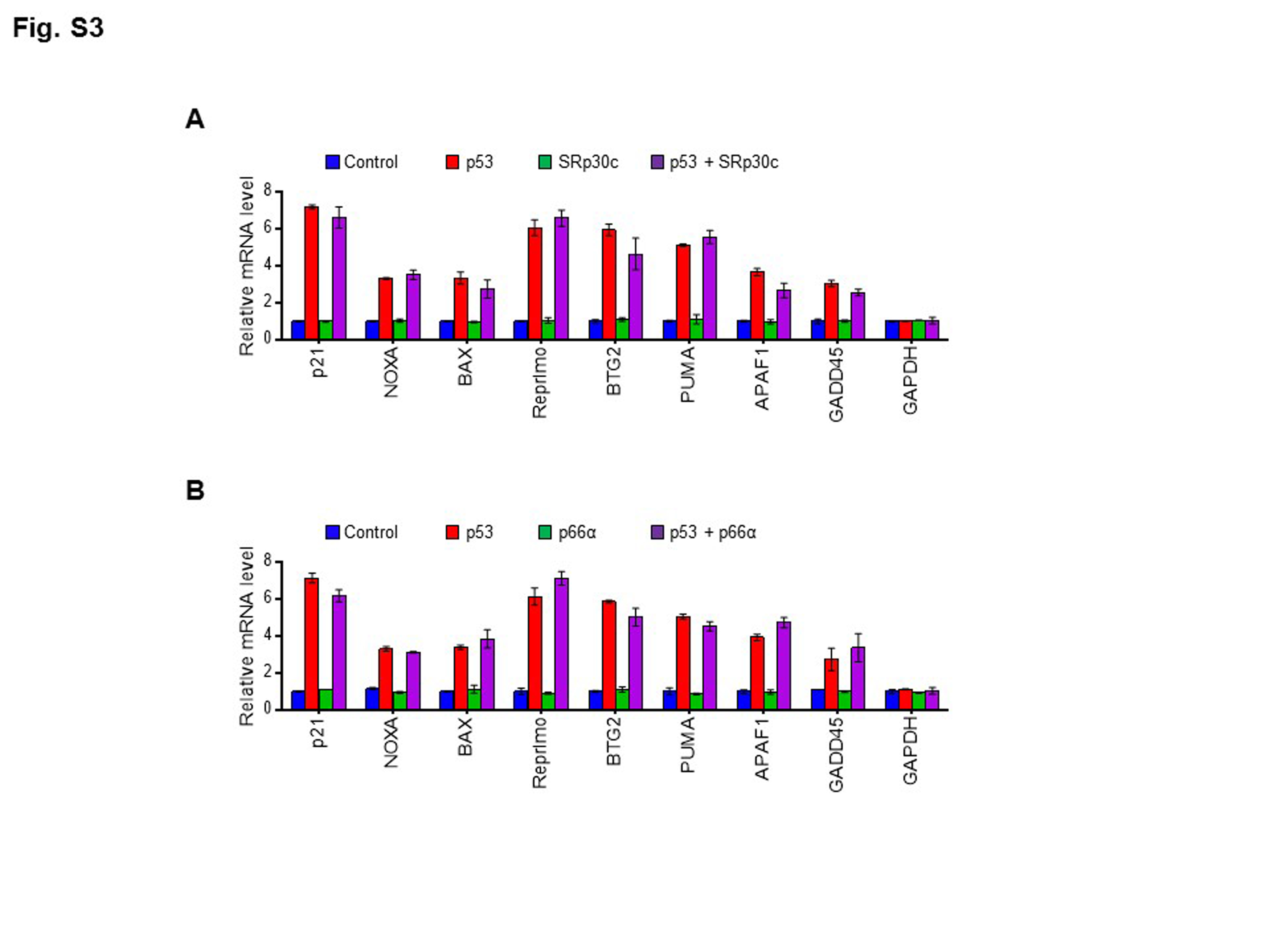

Supplement: Supplementary file 3 — Fig. S3 . Effects of SRp30c and p66α on p53‐target gene expression. [file MOL2-13-1976-s003.JPG]

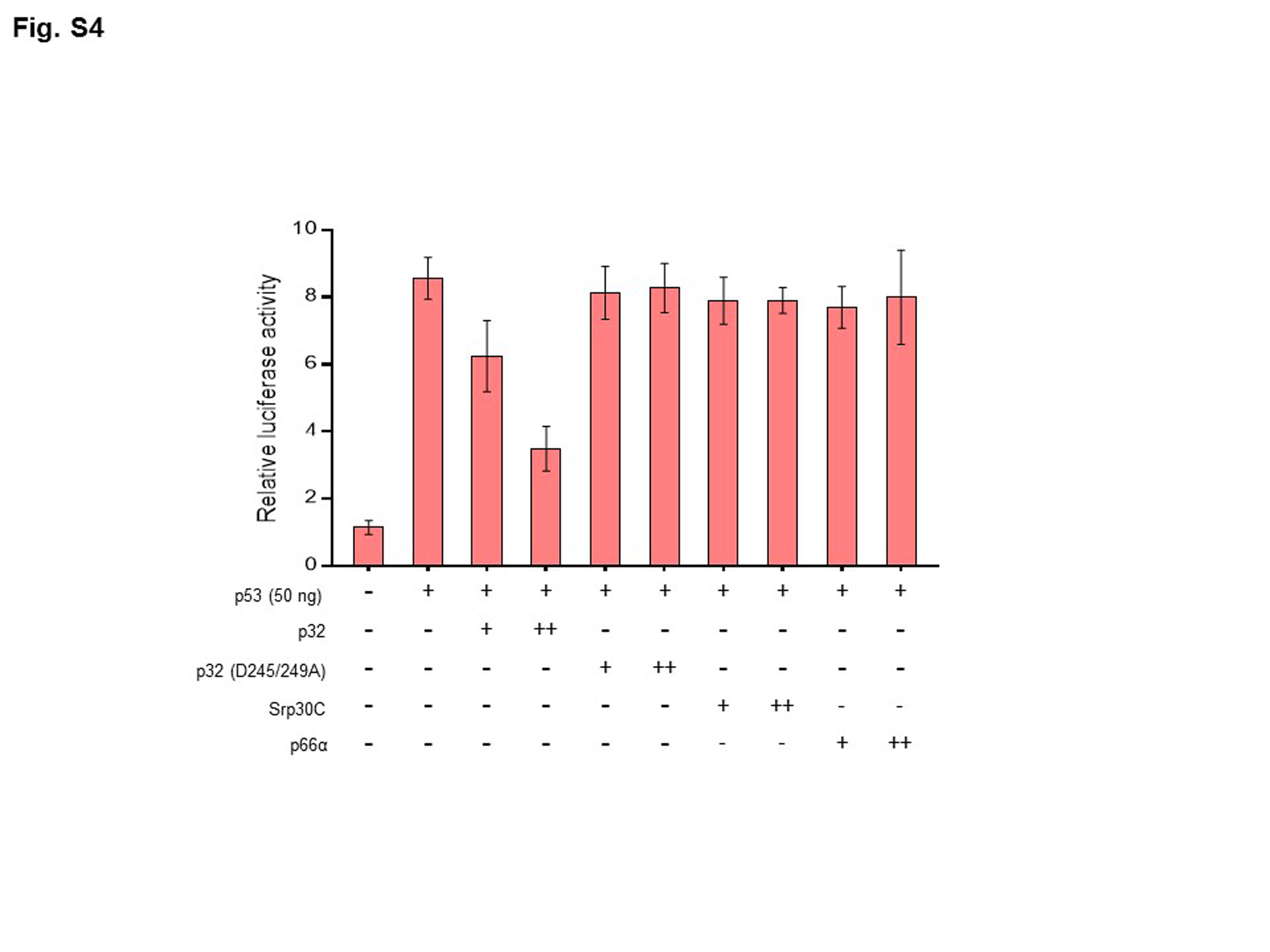

Supplement: Supplementary file 4 — Fig. S4 . p32 interferes with p53 transcriptional activity. [file MOL2-13-1976-s004.JPG]

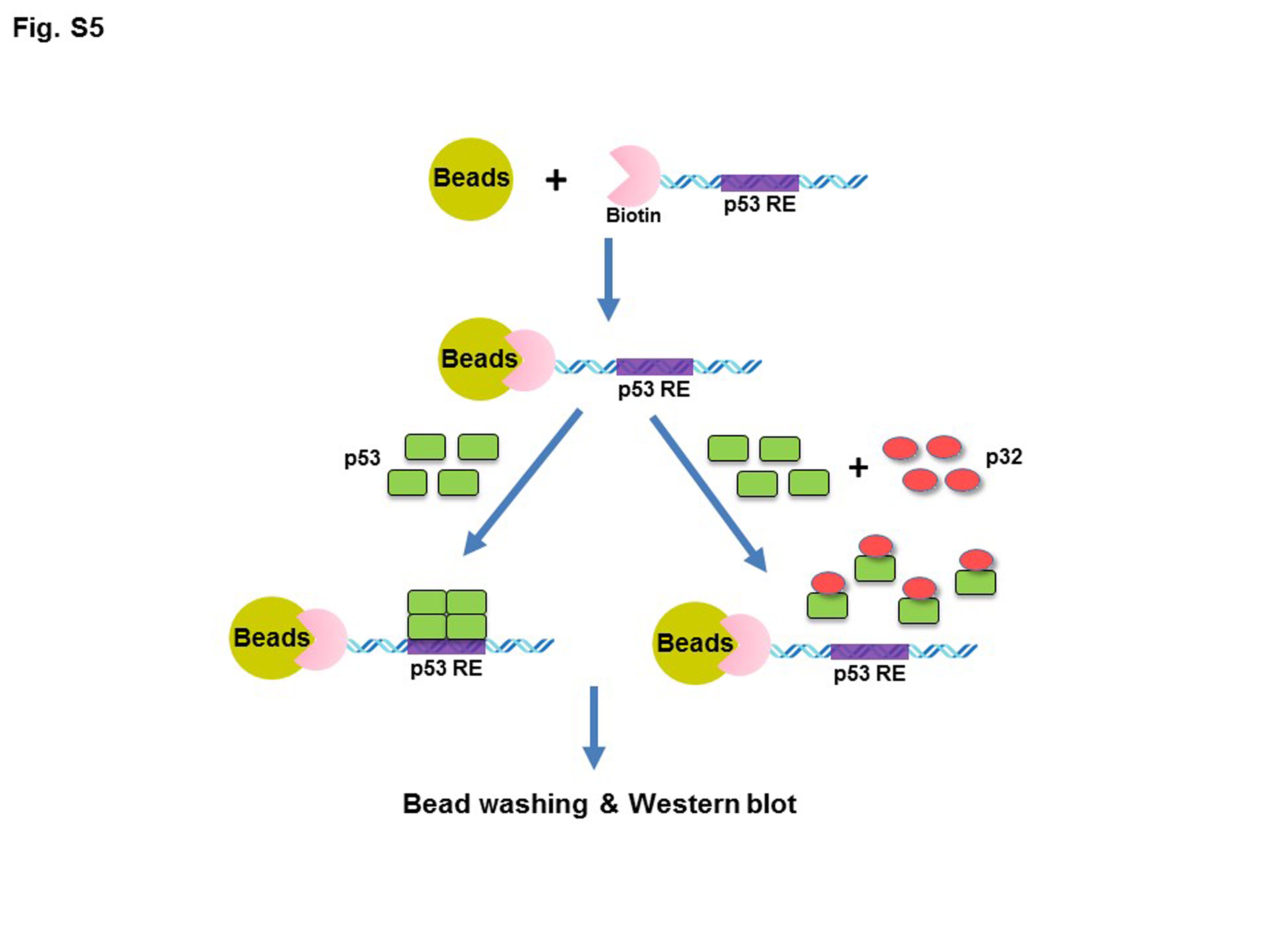

Supplement: Supplementary file 5 — Fig. S5 . Schematic summary of the in vitro DNA binding assay, related to Figs 4A,B. [file MOL2-13-1976-s005.JPG]

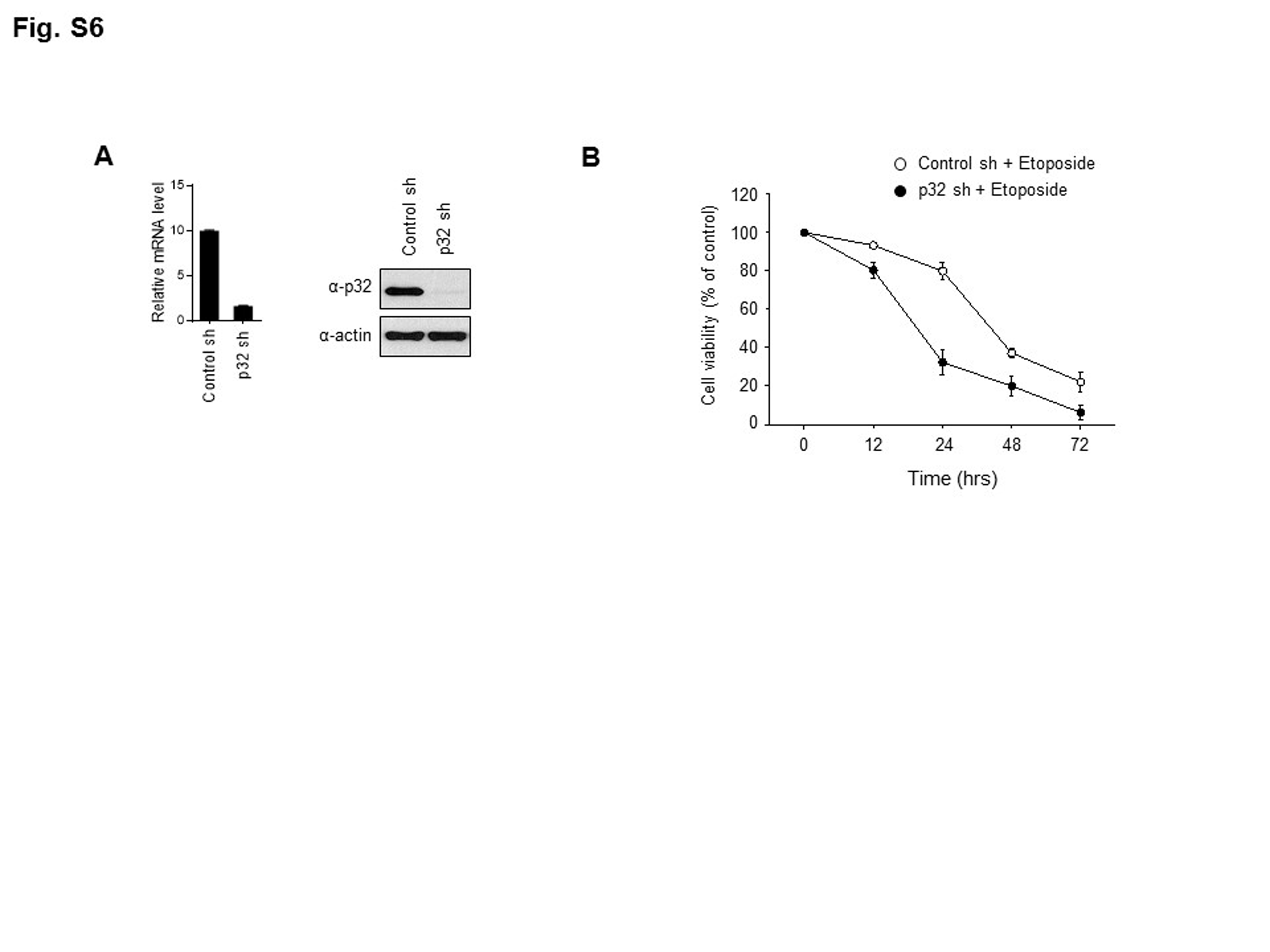

Supplement: Supplementary file 6 — Fig. S6 . (A) p32 depletion in U2OS cells. (B) Effect of etoposide on control and p32‐depleted U2OS cells. [file MOL2-13-1976-s006.JPG]
