# Supplementary material for: p32 is a negative regulator of p53 tetramerization and transactivation
Source: Mol Oncol. 2019 Jul 30;13(9):1976–92. doi: 10.1002/1878-0261.12543 (PMC6717765; doi:10.1002/1878-0261.12543)
Supplement: Supplementary file 7 [file MOL2-13-1976-s007.docx]

**SUPPLEMENTAL INFORMATION**

**p32 is a Negative Regulator of p53 Tetramerization and Transactivation**

Nikhil B. Ghate, Jinman Kim, Yonghwan Shin, Alan Situ, Tobias S. Ulmer and Woojin An

**SUPPLEMENTAL FIGURE LEGENEDS**

**Fig. S1.** List of the factors interacting with wild type (wt) and mutant (mt) H4 N-terminal tails.

**Fig. S2A.** Schematic representation of the *in vitro* transcription assay.

**Fig. S2B.** Western blotting of bacterial purified recombinant proteins. FLAG-tagged p53, p66α and Srp30c were bacterially expressed and purified using anti-FLAG M2 affinity agarose. The purified proteins were analyzed by Western blot with anti-FLAG antibody. Related to Figure 1.

**Fig. S2C.** His-tagged wild type and mutant p32 (74-282 aa) proteins were prepared as described in Materials and Methods and subjected to Western blot analysis using anti-His antibody. Related to Figure 3.

**Fig. S3.** Effects of SRp30c and p66α on p53-target gene expression.

**(A)** H1299 cells were transfected with the expression plasmids encoding p53 and SRp30c for 24 h, and mRNA levels of the indicated genes were measured by RT-qPCR. The results shown are mean values from three independent experiments, and values derived from mock-transfected cells are set to 1. Error bars represent SD.

**(B)** RT-qPCR was performed as in (A), but using H1299 cells transfected with p53 and p66α. The results shown are mean values from three independent experiments, and values derived from mock-transfected cells are set to 1. Error bars represent SD.

**Fig. S4.** p32 interferes with p53 transcriptional activity.

H1299 cells were transfected with p53RE-luc reporter together with expression vectors for wild-type p53 (p53 WT) and p32, Srp30c or p66α as indicated. Cell extracts were prepared 24 h after transfection, and luciferase assays were performed. Data are the mean values ± S.E. of three independent experiments. Values from cells transfected with the luciferase reporter alone are set to 1.

**Fig. S5.** Schematic summary of the *in vitro* DNA binding assay, related to Figures 4A and 4B.

**Fig. S6A.** p32 depletion in U2OS cells.

U2OS cells were transduced with p32-specific shRNA, and knockdown efficiency was determined by RT-qPCR and Western blotting to quantitate expression changes relative to control shRNA.

**Fig. S6B.** Effect of etoposide on Control and p32-depleted U2OS cells.

Control and p32-depleted U2OS cells were mock-treated or treated with 50 µM etoposide for 0, 12, 24, 48, 72 h. Cell viability has been determined using WST-1 cell proliferation reagent. Data represent the means ±SD of three independent experiments. Error bars represent SD.
